# Does Real-Time Feedback Affect Sensorimotor EEG Patterns in Routine Motor Imagery Practice?

**DOI:** 10.3390/brainsci11091234

**Published:** 2021-09-18

**Authors:** Anatoly N. Vasilyev, Yury O. Nuzhdin, Alexander Y. Kaplan

**Affiliations:** 1 Department of Human and Animal Physiology, Faculty of Biology, M.V. Lomonosov Moscow State University, 119234 Moscow, Russia; nuzhdin.urii@gmail.com (Y.O.N.); akaplan@mail.ru (A.Y.K.); 2 MEG Center, Moscow State University of Psychology and Education, 123290 Moscow, Russia; 3 Center for Neurotechnology and Machine Learning, Immanuel Kant Baltic Federal University, 236016 Kaliningrad, Russia

**Keywords:** motor imagery, sensorimotor, EEG rhythm, brain-computer interface, desynchronization, neurorehabilitation, vividness, linear mixed effects, generalized eigendecomposition, open loop

## Abstract

Background. Motor imagery engages much of the same neural circuits as an overt movement. Therefore, the mental rehearsal of movements is often used to supplement physical training and might aid motor neurorehabilitation after stroke. One attempt to capture the brain’s involvement in imagery involves the use, as a marker, of the depression or event-related desynchronization (ERD) of thalamocortical sensorimotor rhythms found in a human electroencephalogram (EEG). Using fast real-time processing, it is possible to make the subject aware of their own brain reactions or—even better—to turn them into actions through a technology called the brain–computer interface (BCI). However, it remains unclear whether BCI-enabled imagery facilitates a stronger or qualitatively different brain response compared to the open-loop training. Methods. Seven healthy volunteers who were experienced in both closed and open-loop motor imagery took part in six experimental sessions over a period of 4.5 months, in which they performed kinesthetic imagery of a previously known set of finger and arm movements with simultaneous 30-channel EEG acquisition. The first and the last session mostly consisted of feedback trials in which the subjects were presented with the classification results of the EEG patterns in real time; during the other sessions, no feedback was provided. Spatiotemporal and amplitude features of the ERD patterns concomitant with imagery were compared across experimental days and between feedback conditions using linear mixed-effects modeling. Results. The main spatial sources of ERD appeared to be highly stable across the six experimental days, remaining nearly identical in five of seven subjects (Pearson’s ρ > 0.94). Only in one subject did the spatial pattern of activation statistically significantly differ (*p* = 0.009) between the feedback and no-feedback conditions. Real-time visual feedback delivered through the BCI did not significantly increase the ERD strength. Conclusion. The results imply that the potential benefits of MI could be yielded by well-habituated subjects with a simplified open-loop setup, e.g., through at-home self-practice.

## 1. Introduction

The voluntary mental rehearsal of body movements (motor imagery, MI) is regarded as an effective tool for increasing sports performance [1] and is often proposed as a form of neurocognitive therapy for cerebral motor deficits, such as those caused by stroke [2]. Systematic reviews [3,4] indicate the effectiveness of MI when used both alone and in synergy with conventional motor-based therapy. Importantly, most reviews [5] on the topic point out the lack of uniformity in the approaches to organizing imagery interventions across different studies. Such heterogeneity could be attributed to the intangible nature of a mental image.

As a strictly mental exercise, motor imagery poses a challenge for practitioners who aim to measure motor imagery quality objectively. One strategy that is adopted for validating and enhancing mental practice is the use of real-time feedback enabled by a brain–computer interface (BCI)—a technology that uses real-time neural activity for the detection (statistical classification) of associated mental states [6]. Commonly, such motor imagery BCIs use a well-known and prominent neural marker of sensorimotor activation, known as the event-related desynchronization of sensorimotor rhythms (ERD of SMR), which is found in a human electroencephalogram (EEG) and represented as a reduction in the amplitude of oscillatory activity, most commonly in the mu (8–14 Hz) and (or) beta (15–30 Hz) frequency bands. Although the sensorimotor ERD is common to a wide variety of sensory, motor and mental events, researchers believe that it can be used to assess the overall engagement of a sensorimotor system in an imagery task [7].

It is important to note, however, that the amplitude of an imagery-related ERD depends on the resting-state SMR amplitude, which is an innate feature of one’s EEG and is unrelated to the imagery ability. This makes the BCI-based monitoring of motor imagery inaccessible for a significant portion of the population [8]. Furthermore, our previous study [9] showed that the amplitude of the SMR-ERD did not predict an increase in cortical excitability concomitant with performing motor imagery, while self-assessed imagery vividness showed an association with an increase in excitability [10]. Taken together with sport psychologists’ practices [11], this evidence suggests that vivid kinesthetic (based on bodily sensation) imagery of a specific movement is responsible for the greatest effect, and an accurate, true-to-life mental representation of a movement should be pursued. The following question remains: does putting a person into a closed-loop BCI to practice imagery help to achieve this goal?

In the classical control-oriented BCI paradigm, the main goal is to achieve a fluent and error-free transfer of information between a brain and detector, which partly relies on the subject’s ability to inadvertently shift their cognitive strategy to whichever approach yields a better classification result [12,13]. This markedly differs from the agenda of the “training BCI”, in which adherence to a certain strategy is paramount. Realizing that researchers have proposed that feedback should more closely match the sensory feedback of a similar performed movement, tactile feedback, virtual animated hands and exoskeletons have been introduced [14,15,16,17]; in other words, making feedback congruent with the performed mental task. This, in turn, transforms the feedback environment into an integral component of the brain’s response (“enriched medium”), arguably shifting motor imagery towards an exogenous stimulus paradigm and making it more difficult to dissociate EEG dynamics related to the endogenous component, i.e., mental effort.

Instead of continuous real-time feedback, delayed discrete feedback is often adopted in training BCIs. Here, reinforcement is delivered on a sparse schedule (every 10 s or once per imagery block), which is far beyond the acceptable delay for brain rhythm modulation paradigms [18] (for more in-depth discussion on neurofeedback modes, readers might refer to [19]). Whereas the above-mentioned feedback strategies could be useful for mental practice, when studying imagery in conjunction with congruent sensory modalities and delayed reinforcement schedules, it becomes much harder to examine the role of the closed-loop component properly, which requires multiple controls, including sham feedback, and is often impractical, especially for clinical research. Therefore, in the present research, we only considered the real-time continuous feedback of visual modality typically used by the BCI/neurofeedback community.

One additional factor that is rarely considered in research is the level of proficiency in motor imagery. It has been shown that the level of expertise in motor imagery greatly affects the involved neural substrates [20,21]. However, the most commonly BCI-assisted imagery is studied in naïve subjects without a differentiation between learning and practice phases, and a proper introduction to the motor imagery technique is often not performed (or not reported; for more in-depth discussion on the importance of an introduction to imagery, see [22]). It seems reasonable to assume that an improper introduction to motor imagery coupled with the immediate exposure to SMR-BCI would reduce training to the form of a mu/beta neurofeedback exercise, defeating the purpose.

To our knowledge, no longitudinal studies of the role of BCI feedback in the regulation of SMR dynamics during motor imagery have been conducted to date. Therefore, the aim of the present study was to evaluate the effect of real-time feedback on the features and within-subject stability of sensorimotor ERD patterns during motor imagery in non-naïve subjects and, thus, to assess the potential increase in training efficiency with a BCI-aided protocol compared to the open-loop design. The study focused on repeated interventions with a familiar set of imageries but also explored the effects of real-time feedback on ERD patterns during the imagery learning (habituation) phase. Additionally, a video-guided imagery protocol is proposed and was explored as an alternative open-loop approach for performing motor imagery.

## 2. Materials and Methods

### 2.1. Participants

Seven healthy volunteers participated in the study (2 women, with a mean age of 26, ranging from 23 to 31 years old). Five participants were right-handed, and two (*s03* and *s05*) were left-handed, as assessed with the Edinburgh handedness questionnaire [23]. All seven subjects had previously taken part in motor imagery studies in our laboratory, where they were first introduced to and taught to perform kinesthetic imagery of hand and arm movements as detailed in [9]. Six subjects of the current study have also participated in [9], their average BCI accuracy was 0.91 (from 0.87 to 0.96) and average ERDd score was 57 (from 44 to 68). The total number of experimental days involving motor imagery tasks prior to the present study ranged from 8 (Subject *s03*) to 64 (*s05*), with a median of 34 days. The experimental procedures were approved by the Lomonosov Moscow State University Committee for bioethics (protocol no. 25-ch). The study followed the Declaration of Helsinki Ethical Principles for Medical Research Involving Human Subjects. All the subjects were informed about the procedures of the study and gave their written consent to participate.

### 2.2. Study Design, Procedure, and Tasks

The study was structured into 6 experimental days that spanned 4–4.5 months (median, 131 days). This rather large time window was selected to check if the subjects would unlearn the motor imagery without feedback as well as to assess the possible temporal deterioration/change of the EEG patterns. All the experimental sessions were conducted under the same conditions (room, experimenter, etc.), predominantly during the early evening (3–7 p.m.). The subjects sat in an armchair in a comfortable, slightly reclined position with their hands either on the armrests (for shoulder movement imagery) or on the hard-surfaced laptop support (Ikea “Byllan”) with their fingertips contacting the surface (for finger movements). The stimuli (pictograms) for conditions requiring static cues were presented on a 24-inch LCD monitor positioned in front of the subject, 1.4 m away from their eyes. For the condition involving continuous video stimulus (TRv; see below) short point of view video clips of the thumb movements were presented on a small, 10-inch portable screen, positioned immediately above the subject’s own hand to match the perspective and location of the hand on the video. Each experiment started with an EEG cap montage, which took 15 to 25 min.

During the experiment, the subjects performed two classes of mental tasks: kinesthetic motor imagery (five variations) or a visual attention task. The tasks were organized in runs (“recordings”) that comprised twelve 6 s trials: six motor imagery trials (1 variation per recording) and six visual attention trials mixed in a pseudo-random order and separated by interstimulus intervals that lasted 2–3 s. During the visual attention task, a picture with complex geometrical shapes appeared on a screen. The subjects’ task was to silently count any elements of the picture (angles, lines, dots or protrusions) at their own comfortable pace. This condition was a reference non-motor state for all the types of imagery and served multiple purposes. Firstly, on a psychological level, this task helped the subjects to “switch” from mental imagery, requiring a comparable level of attention to perform it, and prevented the participants’ minds from wandering. Secondly, this task provided us with a stable and reproducible cognitive state, as it was familiar for subjects; note that this reference task was interlaced with the imagery trials that had to be compared with it. Thirdly, the visual attention caused the depression (ERD) of occipital alpha rhythms, while sensorimotor rhythms reacted in the opposite way, providing a good functional separation of sensorimotor and visual brain networks, thus serving as a functional localizer task.

During the motor imagery trials, a pictogram with a left or right hand/arm appeared on the screen, serving as a cue for a subject to perform motor imagery of one of the five following movement types. Finger imagery (FR for right and FL for left hand finger imagery) corresponded to a random order of two to five individual finger presses (flexion at the metacarpophalangeal joint). Shoulder imagery (SR for right and SL for left shoulder imagery) corresponded to a forward/backward circumduction from a seated arm-on-armrest position (referred to by the subjects as the “crawl stroke”). Right hand thumb swipe imagery (TR) corresponded to a movement mimicking sliding one’s thumb across a smartphone display (from the lower left to upper right corner). For this movement, we designed an ergonomic hand stand, which supported the palm in a neutral position and provided a smooth surface for thumb movement. The subject’s hand was inserted into the stand for the trials requiring thumb imagery. The thumb swipe imagery was performed in two variations: with an external pictogram-cue (similar to other movements and further denoted TR) and with a video stimulus, guiding the subject’s mental movements (TRv). In the latter mode, a subject had to kinesthetically imagine their thumb movements, mentally reproducing the phases and speed of movements on the video in real time. The goal of the TRv condition was to compare other TR variations (without and with feedback) in terms of the reduction in mental load while providing additional sensorimotor brain activation through visual congruency, yet without introducing a cumbersome feedback loop. Outside the TRv condition, all the imagined movements were performed in a self-paced mode at random intervals between movement phases (taps of individual fingers or forward/backward shoulder strokes). Both the TR and TRv movements were novel to the subjects and were performed only during Days 5 and 6 of the present study, while the other imagery types were thoroughly practiced in a similar environment during previously attended studies. The technique for performing the kinesthetic imagery of self-paced and self-randomized movements was also known by all the subjects; please refer to [9] for details.

**Figure 1:**
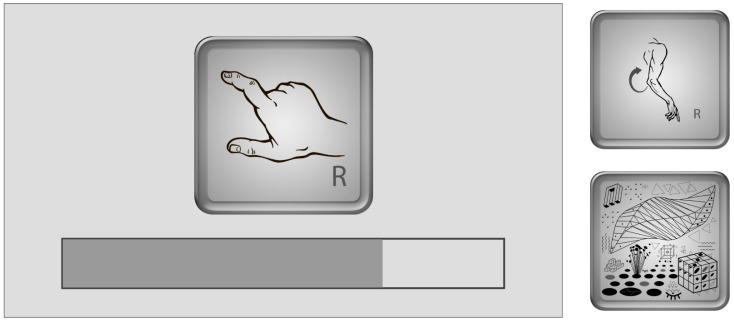
(**left**) Stimulus presentation display with the feedback progress bar. The correct classification of the target mental state (in this case–FR) caused the “progress bar” animation to play. (**right**) Pictures used for cueing shoulder imagery (SR) and the visual attention task.

The experimental runs were organized in blocks of two to three recordings with the same type of imagery (e.g., FR, FR, FR, FL, FL, etc.). The FR condition was prioritized, and twice the number of runs of other types was performed (typically six runs per day for FR and three runs for the other types). Based on the goals of the study, the experimental days (sessions) were divided into days with BCI assistance (with feedback) and those without feedback (cued imagery only). The first and the last session (No. 1 and 6) were BCI-assisted, while the sessions between these (No. 2, 3, 4 and 5) were without feedback. This design was selected to examine whether the subjects would gradually “forget” how to perform imagery with no feedback. Continuous feedback was presented to the subjects during a mental task in the form of an animated bar displayed immediately below the cue pictogram (Figure 1). At the beginning of the trial with feedback, an empty box appeared below the pictogram; starting from 1.5 s relative to the cue onset, the correct classification of the target mental state caused the “progress bar” animation to play. Subjects were asked to “fill the bar” as much as they could, with 100% corresponding to the correct classification of all the time intervals within the trial. No questions from the subjects about their performance (the “EEG rhythm’s strength”) during the study were answered.

### 2.3. Signal Acquisition and Processing

The EEG was recorded using an NVX52 DC amplifier (MKS, Zelenograd, Russia) with 30 passive Ag/AgCl electrodes covering the sensorimotor cortex area according to the “10–10” international system in the following positions: F3, F4, FC5, FC3, FC1, FCz, FC2, FC4, FC6, C5, C3, C1, Cz, C2, C4, C6, CP5, CP3, CP1, CPZ, CP2, CP4, CP6, P5, P3, P1, PZ, P2, P4 and P6. The reference electrode was in position TP10. The electrode–skin impedance was kept below 20 kΩ. The signal was sampled at 500 Hz with a 50 Hz notch filter. For offline analysis, the signal was filtered using a zero-phase bandpass FIR filter with cutoff frequencies of 3 and 48 Hz.

The data acquisition, stimulus presentation and feedback delivery were performed using the BCI2000 [24] software with a custom classifier module. Custom MATLAB scripts were used to generate classifier weights based on the learning dataset comprising data previously recorded on the same day, expanding as far as four recordings relative to the moments of the weight recalculation. The classifier weight calculation was based on the following algorithm, which included four steps: bandpass filtering in 6–40 Hz using a fourth-order Butterworth filter; calculating spatial filters through the generalized eigendecomposition (GED) of multichannel EEG signal covariance matrices corresponding to motor imagery and visual attention trials (also known as common spatial patterns [25]); extracting the time–frequency by performing a short-time fast Fourier transform with a 1 s window and 90% overlap; and training the naïve Bayes classifier on power features corresponding to the five spatial filter–frequency combinations with the most divergent power distributions. For more details, please refer to [9].

### 2.4. Data Analysis

We focused our data analysis on three features of the motor-imagery-related EEG signal: the spatial patterns, strength of the sensorimotor rhythm ERD, and BCI-classification performance. In order to take advantage of the multichannel EEG signal while avoiding the electrode-wise mass univariate analysis, we aimed to extract a set of spatial components, or “sources”, related to the SMR-ERD reaction. It should be noted that the term “source” is used here in a strictly statistical sense—i.e., as directions or subspaces within a 30-dimensional sensor signal space, without considering the underlying neuroanatomy. To identify ERD sources, we considered the spatial directions in which the signal in the SMR frequency band (mu + beta, from 7 to 27 Hz) had the least variance in the motor imagery condition (sensorimotor active ~ desynchronization of SMR) while simultaneously having the most variance in the visual attention condition (sensorimotor passive ~ synchronization of SMR). This task could be solved by the generalized eigendecomposition [26] of signal covariance matrices corresponding to motor imagery ($C_{active}$) and visual scene ($C_{passive}$) written in the forms of Equation (1) or Equation (2).

(1) $$\Lambda =\mathrm{argmax}\left\{\frac{W^{T}C_{active}W}{W^{T}C_{passive}W}\right\}$$

(2) $$C_{passive}^{-1}C_{active}W=\;W\Lambda$$

where W is the matrix containing eigenvectors in its columns and Λ is the diagonal matrix with elements on the main diagonal, which are eigenvalues corresponding to eigenvectors in W.

After calculating W, for ERD sources, we considered only columns (spatial filters) corresponding to the smallest Λ, i.e., with minimized $\frac{C_{active}}{C_{passive}}$. Following that, a forward model A was computed by passing W^T^ through the covariance of the active state: A ∝ W^−T^ ∝ W^T^$C_{active}$. Several measures were taken to prevent the contribution of signal noise and the overfitting of W. Firstly, the trial covariance homogeneity was enforced by rejecting trials based on the Euclidean distance (>2SD) from their covariance matrix to the average (median) covariance matrix of all runs (within days and runs of the same condition). Secondly, we used 10-fold cross-validation, performing GED on the covariance, computing over 90% of the analyzed signals and applying it to the rest of the data. The average (mean) forward models of selected sources were compared across experimental days using Pearson correlation coefficients.

After calculating and applying spatial filters, we performed time–frequency decomposition using a short-time Fourier transform (1 s windows, Hann function, and 90% overlap). Then, for each frequency bin (1 Hz resolution) and each spatial component, we computed the normalized power distributions for the motor imagery condition and visual attention task (for both conditions in 2 to 6 s intervals relative to the cue onset) and calculated the distribution overlap (as a percentage)–Equation (3).

(3) $${\mathrm{ERD}}_{d}=100\;*\;\frac{1}{2}\overset{\infty }{\underset{0}{\int }}\left|f_{active}\left(p\right)-f_{reference}\left(p\right)\right|\;dp$$

where $f_{active}\left(p\right)$ and $f_{reference}\left(p\right)$ are the probability density function estimates for the SMR power (p) in the active (imagery) and the reference conditions (visual attention) correspondingly, with $\int_{0}^{\infty }f\left(p\right)dp=1\;$, and |.| indicating the absolute value operator.

The resulting score was called the ERDd (d—for distribution analysis) and was used to quantify the ERD strength. The ERDd score is negative when the power distribution of an active condition has a median lower than that of the reference condition, and positive otherwise. Thus, stronger desynchronization corresponds to more negative values, and synchronization, to positive values. In contrast to the commonly used average power amplitude difference, ERDd makes no assumptions about the shape of the power value distribution and is better suited to quantifying a steady-state process, such as the ERD, concomitant with continuous sensorimotor events. Readers may refer to [9] for a graphical explanation of the ERDd and its comparison with conventional metrics.

The ERDd scores calculated for each frequency bin and each spatial component were used to identify a set of user-specific spatio-spectral features through the following procedure. First, the components with the largest absolute ERDd scores within 7–26 Hz bins were selected. Then, neighboring frequency bins with the score above 30% of the largest were picked to form a frequency range. Average of the Fourier coefficients for these ranges was used later to recalculate the ERDd and to train the classifier. Components with the largest negative ERDd (closer to −100) later referred to as the “strongest ERD sources” typically corresponded to the first two to five columns of W in Equation (1), revealing the SMR ERD sources. Conversely, components with the largest positive ERDd (closer to 100) corresponded to the last columns in W and usually pointed at the occipital event-related synchronization (ERS), reflecting reduced visual information processing during motor imagery. While the rest of the paper mainly concerns the SMR ERD sources and features, an addition of ERS-based features could provide better classification accuracy and therefore was investigated using the ERS+ERD classifier (see below).

To estimate the performance of the BCI for the trials with and without feedback, we performed two-fold cross-validation five times using the same classification algorithm as that used online. The data from runs of the same motor imagery condition during the particular experimental day were concatenated in one block for cross-validation; thus, the calculated accuracy estimates corresponded to day × condition, without differentiation into recordings. The classification accuracy was estimated using two sets of classifiers: one using only ERD features and another using the best of all the available features (including the occipital alpha ERS, for example). In addition to the five iterations of two-fold cross-validation, we performed accuracy estimation using the time-series cross-validation, where the training set varied in size and was composed of all the previous trials related to the trial under consideration. The latter method served as a more realistic approach for estimating accuracy in the experimental session, since it uses only past data for the classifier training.

The statistical inference of spatial patterns was performed using Pearson correlation coefficients. We calculated the correlation matrix of the mean forward models for six experimental days, then averaged the correlation between the patterns of all the feedback days and non-feedback days and subtracted this value from the mean correlation among the patterns on non-feedback days. Then, we repeated the procedure while permuting “feedback” labels 100,000 times, randomly assigning these labels to any two sessions. Using the post-permutation statistical distribution, we calculated the *p*-value for the value observed with the true labels. A left-tailed (lower correlation in the feedback condition) *p* = 0.05 threshold was used to assess significance.

As the ERDd value was available for each experimental run (recording), we were able to use a linear mixed-effects model to capture the intraday and interday variance. We used the mean ERDd score of the two strongest sources (commonly corresponding to the ipsi- and contralateral SMR sources) as a response variable and modeled it with fixed and random effects depending on the effects in question (Equations (4a,b)–(6), [27]). The MATLAB function *fitlme* was used to fit the data using the restricted maximum likelihood (REML) method to estimate parameters. The statistical significance was evaluated for fixed effects using the *anova* function with Satterthwaite approximations for the degrees of freedom (resulting in a fractional number for df) [28].

Firstly, we tested if the number of days affected the ERD strength (Equation (4a,b)), modeling the gradual deterioration of the brain response in the absence of feedback (Days 2–5). The statistical significance of the fixed effects of the “day” and the day–imagery type interaction was assessed using ANOVA. Then, models with and without a random “day” and fixed effects were fitted using the maximum likelihood (ML) method and compared using a simulated likelihood ratio with 1000 simulations. The latter was performed to asssess the significance of the “day” factor and thus reject/include it in further analysis.

ERDd ~ 1 + day * *imageryType* + (1 + day | *subj*) (4a)

ERDd ~ 1 + *imageryType* + (1 | *subj*) (4b)

Secondly, we modeled the binary “feedback” *(“isFeedbacked”)* factor and its interaction with the “subject” *(“subj”),* as well as the random effect *imageryType* and intercept for the grouping variable of the “subject” (Equation (5)). For this and the previous model, we used data from trials with familiar imageries (conditions FL, FR, SL and SR).

ERDd ~ 1 + *isFeedbacked* * *subj* + (1 + imageryType | *subj*) (5)

Thirdly, to estimate the effectiveness of the alternative imagery mode with the video stimulus, we used a model encompassing only data with newly introduced imagery (conditions TR and TRv) and fitted the model with the categorical factor *videoORfeed* (TR + no feedback, TRv, TR + feedback). Furthermore, the TRv and TR + feedback levels were assessed to compare the two approaches directly.

ERDd ~ 1 + *videoORfeed* + (1 | *subj*) (6)

A two-way ANOVA was performed on the classifier’s output values using the factors “subject” and “feedback” to assess the differences in the accuracy of the BCI between subjects depending on the presence of feedback. For all the above-mentioned tests, alpha was set at *p* = 0.05.

## 3. Results

### 3.1. Spatial Patterns

**Figure 2:**
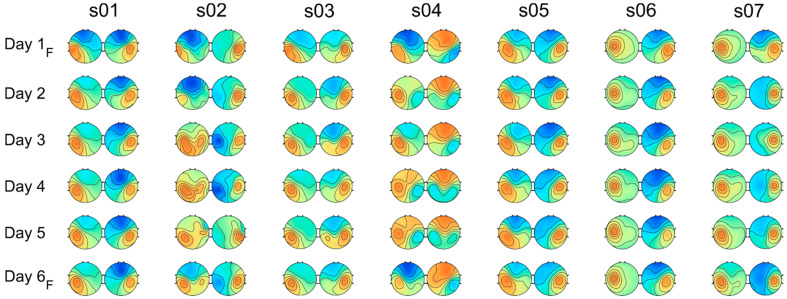
Forward models of the most significant SMR-ERD sources for motor imagery of right-hand fingers. For all subjects, contralateral and ipsilateral-dominant sensorimotor desynchronization sources were found and showed the largest desynchronization amplitudes. Colors correspond to the relative weights of EEG channels (red for positive and blue for negative weights; color scale is linear and symmetric). Spatial filters (eigenvectors) for corresponding components were sign-corrected to match directions across all six experimental days. For Days 1 and 6, models for feedbacked trials are shown (denoted by “F”).

Spatial filters for the ERD sources and corresponding forward model estimates were computed using GED using 10-fold cross-validation. Forward models of the two strongest ERD sources (corresponding to the smallest λ, Equations (1) and (2)) for all the subjects and experimental days were almost always symmetrical with strong ipsilateral or contralateral dominance (except for 7 of 84 times, when the ipsilateral source was the third most dominant source). Figure 2 shows the patterns for the FR condition organized in pairs for each experimental day and subject. Note that, even with the inherent imperfection related to the manual installation of the EEG cap, the spatial patterns show distinctive asymmetries relative to the AC–PC line, which might be caused by anatomical variations in the Rolandic region [29].

Using the permutation (N = 100,000) of “feedback” labels for different days independently for ipsilateral and contralateral sources, we tested whether their forward projections differed according to the feedback condition. Only one subject, *s04*, showed a statistically significant difference (*p* = 0.009) in their pattern related to the feedback condition. Note that, on the feedback days (Days 1 and 6), the sources conformed to the tangential dipolar structure, while on days without feedback, radial and bilaterally smudged patterns prevailed. The spatial patterns of *s02* were not significantly different in feedback conditions but showed, similarly to *s04,* rather high day-to-day variations in contralateral sources. The mean Pearson’s correlation coefficients for the contralateral sources among the days for *s04* and *s02* were 0.62 and 0.79, respectively, whereas for the rest of the subjects, the mean ρ stayed within the interval of 0.95–0.99.

### 3.2. ERD Strength

**Figure 3:**
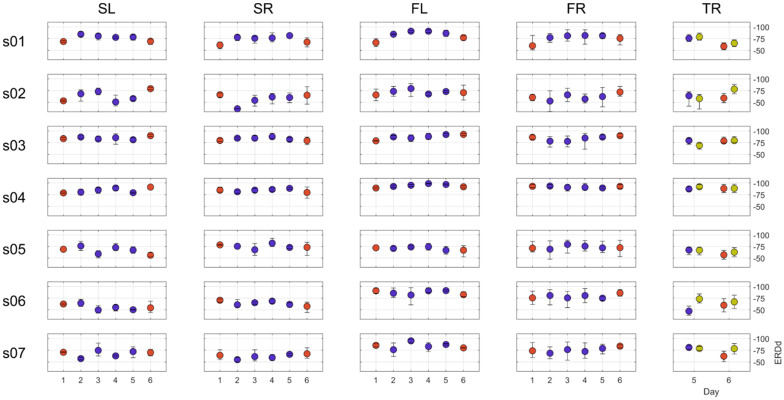
ERDd values corresponding to different motor imagery conditions for six experimental days. Event-related desynchronization strength measured using the ERDd score (OY axis); each point represents the mean value across all runs during a particular experimental day (OX axis), and error bars correspond to min–max range. For Days 1 and 6, only feedbacked runs were taken. Columns are motor imagery conditions: SL(R)—left (right) shoulder movement, FL(R)—right hand finger movement, TR—right hand thumb swipe. Color of the data point corresponds to the feedback condition: red—continuous visual feedback, blue—no feedback (static pictogram stimulus), and green—no feedback (continuous video stimulus).

The event-related desynchronization strength was quantified using the ERDd score, representing the non-overlapping area between the probability distributions of the SMR power during the imagery and visual attention tasks [9]. The average ERDd values for every imagery type, day and subject are presented in Figure 3. Note that the data point diameter was approximately 12% according to the ERDd scale; therefore, if error bars (min–max) are not visible, they are within 12 points. Note that, for most of the conditions and subjects, the ERDd values showed low intraday variability while clearly differing between subjects. Subject *s02* showed the most variability (especially in the shoulder imagery) and had the lowest mean values across all the conditions.

To estimate the possible gradual deterioration of the ERD response in the absence of feedback (from several weeks (Day 2) up to four months (Day 5)), the effects of the day and the day–imagery type interaction were tested using the model from Equation (4a). No significant group effect was found for the day—F_(1, 342.26)_ = 1.9855, *p* = 0.15972—and the day–imagery type interaction—F_(3, 409.02)_ = 2.0686, *p* = 0.10378. Following that, two models were fitted using the ML method: a more complex model (Equation (4a)) with df = 12 and a simplified model without the term “day”, both in fixed and random parts of the model (Equation (4a)) with df = 6. Those two models were then compared using a simulated likelihood ratio with 1000 simulations, which revealed no statistical advantage of a more complex model: the likelihood ratio test statistic was 9.202, and the simulated *p* was 0.13187 with a 95% confidence interval for a simulated *p* ranging from 0.11152 to 0.15441. For that reason, the “day” factor was excluded from further analysis.

Next, to assess the effects of feedback on the SMR-desynchronization strength, we fitted mean ERDd values for the two most prominent sources with a linear mixed-effects model and introduced and tested the group effects of factors of interest (Equations (4a,b)–(6)). Testing the group-level effect for *isFeedbacked* revealed no statistically significant results—F_(1, 611.4)_ = 2.9479, *p* = 0.086495—while the individual effects for *isFeedbacked*:*subj* were quite varied (Table 1), with a single strong statistically significant result for Subject *s01*—tStat = 6.6879, *p* = 5 × 10^−11^; for that subject, feedback increased the ERDd value (BLUE: 13.386, CI95%: 9.46–17.32), corresponding to weaker desynchronization.

The analysis of the ERD patterns of the novel movement type (TR or TRv) introduced in Sessions 5 and 6 yielded statistically significant differences among the approaches of imagery conditions—F_(2, 75.203)_ = 4.1636, *p* = 0.019278—with the ERD strength increasing from feedback to no feedback to video stimulus conditions. This result shows that the imagery condition does impact the ERD strength when learning to perform new imagery. When comparing the feedback and video stimulus conditions, the latter condition was found to induce a stronger ERD: F_(1, 75.203)_ = 8.3263, *p* = 0.0051.

**Figure 4:**
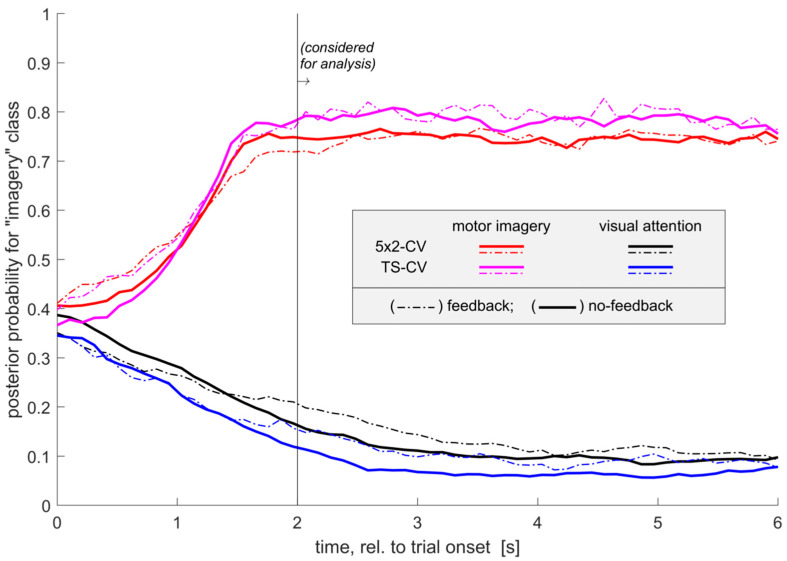
Group-average time courses of Bayes classifier output using SMR-ERD features for condition FR. Two cross-validation schemes are presented: the “5 × 2” scheme used an equal amount of data for training and testing across all folds; the “TS” scheme used a progressively expanding training set encompassing all past trials within a particular experimental day.

The theoretical classification accuracy was estimated for all the days with the same classification algorithms used online, employing “5 × 2” and “time-series” cross-validation schemes (see Section 2). The average accuracy in a [2 to 6] s window was used, and the group-averaged time course of the classifier output is presented in Figure 4. Two types of classifiers were tested: one with ERD features only (the same as in Sessions 1 and 6) and one with ERD/ERS, with the latter having the advantage of employing such events as alpha-rhythm synchronization or possible focal beta-ERS. Only the results for FR are presented below, as no differences in the hypothesis test results were observed for the other conditions. For the 5 × 2 cross-validation scheme for both types of classifiers, the “feedback” factor was statistically insignificant (ERD + ERS: F_(1, 34)_ = 0.31, *p* = 0.5837; ERD-only: F_(1, 34)_ = 0.5244, *p* = 0.5244), while the “subject” factor was significant (ERD+ERS: F_(6, 34)_ = 5.88, *p* = 0.0001; ERD-only: F_(6, 34)_ = 13, *p* = 1.374 × 10^−7^). According to both ANOVAs, Subject *s02* exhibited lower classification accuracy, most noticeable in the ERD-only classifier, which was in line with the weaker ERD and highly variable spatial patterns reported earlier (Figure 2 and Figure 3). The same statistical conclusions were reached when using the time-series cross-validation scheme. Notably, the time-series approach achieved higher accuracies for both classes and feedback conditions, most likely due to capturing within-session ERS/ERD pattern evolution (three-way ANOVA with the factors “subject”, “feedback” and “cv-scheme”, with the last showing the values F_(1, 75)_ = 19.01 and *p* = 4.076 × 10^−5^).

## 4. Discussion

### 4.1. Feasibility of Instrumental Assessment

In the present study, the term “motor imagery training” is understood in the context of a neurocognitive exercise and not in a sense of teaching or improving the imagery skill, even though practice might and often does lead to improvement. Imagery is an exercise that is usually performed to achieve some improvement of an existing motor skill or in an attempt to reactivate a damaged motor circuit—for example, when impacted by stroke [2,5,30]. Put simply, motor imagery effects are mediated through targeted sensorimotor activation. The precise nature of the activation caused by imagery is outside the scope of the current discussion, especially as the topical literature is far from being in agreement on which particular activation is the most effective to track—for example, for the purposes of motor rehabilitation [31].

It is safe to assume that there would be no difference in the ERD patterns between those caused by the imagery of an index finger flexion and the imagery of an index finger extension, especially in the context of a single-trial detection in BCI. However, as shown by multiple studies [32], imagery demonstrates extremely high specificity to the muscles involved in the corresponding motor act. Therefore, for now, BCIs based on ERD, or based on any of the non-invasive EEG features, cannot be used to control for imagery movement types. While it is theoretically possible that the proper imagery could be controlled using TMS mapping/probing, the authors are not aware of such attempts. One reason for the low feasibility of TMS control is the high variability of motor-evoked responses in combination with the excitability changes caused by a variety of physiological and non-physiological factors [33]. Therefore, for now, the most efficient way of enforcing the proper form of imagery is through instruction supplied by a doctor or imagery instructor, followed by the regular verification of the understanding of the instruction by performing an interview [34].

### 4.2. Alternative to Feedback Need Not Be Nothing

One motivation for the current study was to contest the premise of a group of SMR-BCI studies’ designs with a passive “no-feedback” group with a reported way to work with variable SMR patterns and low ERD values compared to those observed in our previous work. Let us consider the design and results of the multi-day study resembling our present work [35], which reported an advantage of BCI over the no-feedback condition. The authors reported an ERD increase after 5 days of motor imagery BCI interventions compared to the “no-feedback” group. Moreover, a significantly increasing ERD was found for the series “BAR”-feedback (the same progress-bar animation as in the current study) and then “incongruent hand animation”, followed by “congruent hand animation”. According to the imagery instructions, the authors reported that participants “were asked to imagine grasping with their right hand”. The reported percentage value for the best-performing group (CONGRUENT) was 32% for ERD and 76.85% for BCI accuracy, corresponding to approximately 3.35 dB of ERDdb and to 77–80% in terms of BCI accuracy. As we can see, the BCI accuracy in our two studies was close to the corresponding ERD scores; however, in our research, the discussed values were below the 10th percentile, while in the work by Ono et al. [35], it was the mean of the best-performing group. Even when factoring in the difference in EEG setups, a very low ERD strength in the “no-feedback” group in the study by Ono et al. [35] may indicate that their subjects were not performing motor imagery properly, and the same might be concluded, to a lesser degree, about the other feedback groups.

Thus, we speculate that the combination of three possible factors could explain the effects reported by that (or a similar) study: an improvement of the imagery technique accelerated by the ERD-based feedback, a more general instrumental conditioning promoted by the neurofeedback and a stimulation through movement observation (in conditions with hand animation). One way of estimating the proportion of those effects would be to conduct a no-feedback follow-up intervention after the last session for each group (which is similar to the approach taken in the current study). Even if most of the ERD gain in the feedback groups was due to the imagery technique improvement, the conclusion of Ono’s study might be that subjects with no instruction and no BCI feedback cannot learn imagery on their own simply by trying, while subjects with ERD-based BCI could improve the imagery technique by practicing with the feedback.

Similarly designed studies with an explicit comparison of BCI and open-loop imagery interventions [30] conducted on post-stroke patients demonstrated that subjects who received BCI training showed more improvement both in ERD strength and in functional motor rehabilitation. However, the same problem remained: the no-feedback groups received very few imagery technique instructions and thus did not perform their tasks correctly.

Although all our subjects in the current and previous studies received some BCI training, which might have contributed towards shaping their imagery technique, we still strongly suspect that teaching motor imagery through a thorough explanation and discussions with the trainee/patient is vastly preferable to neurofeedback; firstly, because the former approach shapes a correct and verifiable mental strategy, and secondly, because it allows trainees to focus their attention on imagery reflection, thus making imagery more controllable regardless of external stimuli, such as ERD-based feedback.

### 4.3. Open-Loop Versus Closed-Loop Imagery

The vividness of imagery plays an important role in its potential effectiveness in rehabilitation and sport [36]. The purpose of a closed-loop motor imagery practice is thus to modify or help to sustain a subject’s optimal mental strategy using SMR-related features of a person’s EEG. In [37], the authors investigated the effects of spontaneous mental strategies on the performance of SMR upregulation neurofeedback. The authors found that subjects who adhered to some mental strategy performed poorly and showed no improvement throughout the experimental sessions. On the other hand, participants who did not report sticking to a mental strategy by the end of the study displayed improvements in their performance with time, which was most likely due to the development of automatic regulation mechanisms.

In the study by Kober et al. [37], as with most neurofeedback studies, the subjects received no or very minimal and vague starting instructions and had to search for an effective strategy with trial and error. Once they found the effective strategy, automatization could begin to achieve better neurofeedback performance. Conversely, in our current study, the subjects had to learn and follow a specific set of instructions, enforcing kinesthetic imagery. Moreover, our imagery strategy implied mindfulness and, in one sense, withstood automatization. Thus, in a closed-loop BCI, our subjects had to negotiate the guidance of internal and external feedback represented by the vividness of the imagery and SMR-dependent animations, respectively.

Our own [9] and other authors’ studies [38] previously did not report any associations between self-assessed imagery vividness and SRM-ERD or BCI performance. However, in [39], the authors showed that subjects who both never received SMR-based feedback on imagery and were not specifically trained to imagine movements could predict their expected BCI performance based on open-loop practice experience. The latter result suggests that the internal sense of quality and/or controllability of a mental image translates into more expressive SMR activation. Unfortunately, Ahn et al. [39] performed only offline BCI accuracy estimation, whereas online testing may have revealed a decrease in SMR-ERD, similar to that for our subject *s01.*

One would expect that increased mental load while receiving external real-time feedback may cause the deterioration of both the form of internally controlled imagery and BCI performance. One approach to decreasing cognitive load is to make the feedback congruent with the imagined movement, e.g., by controlling an artificial phantom limb or orthosis. However, it would be impossible to achieve movement congruence, as the phases of imagined movement would not match those of the external limb. In our study, we tried to solve this problem with “video-guided” imagery. Our approach aimed to decrease the mental load of imagery by providing video instructions, thus relieving the subject from the duty of self-generating motor sequences. Congruent video stimuli also had an additional benefit of action observation to enhance sensorimotor circuit activation [40,41]. However, recent studies have shown that action observation does not directly involve motor circuits, as is evident from the lack of a gain in cortical excitability [42]. It should also be noted that the benefits from additional congruent stimulation may be outweighed by the potential dependency of subjects’ imagery skill for external stimuli, which could be addressed in further research involving fully naïve subjects.

Motivation is the one important yet difficult-to-quantify advantage of performing routine imagery in a closed loop, especially in a clinical setting. However, we would argue that short-lasting amusement from a BCI-enabled game or device would contribute little to the end goal of mental practice, whereas sustainable motivation born from a goal-orientated mindset may indeed improve imagery efficiency [43].

Summing up the discussed points, we can conclude that SMR-ERD patterns concomitant to kinesthetic motor imagery performed by trained subjects remain stable over long periods of time. This shows that sensorimotor brain activation during motor imagery may be sustained during repeated interventions based only on internal sense image vividness and does not improve with the addition of a real-time feedback loop. With proper training and motivation, motor imagery practice could be fully accessible for patients requiring home-based training [44] and could also be performed in an unsupervised manner [45].

## 5. Conclusions

The goal of the current study was to determine if the mental practice efficiency in experienced users could be enhanced by using a sensorimotor rhythm-based brain–computer interface compared to the open-loop training. To that end, we conducted six motor imagery training sessions that spanned four to five months, in which subjects performed the cued and continuous kinesthetic imagery of hand and arm movements. We found that both the SMR-ERD amplitude and the performance in the motor imagery BCI did not deteriorate with time, and the corresponding scores varied within user-specific ranges. The spatial patterns of ERD, extracted using the generalized eigendecomposition of covariance matrices, also remained stable across the experimental days. Continuous real-time visual feedback did not significantly affect the strength of the ERD at the group level, with a significant negative effect found only in one subject across all the imagined movement types. In summary, this evidence suggests that, in well-habituated subjects, motor-imagery-related ERD patterns remain stable regardless of the presence of real-time feedback, in turn implying that the potential benefits of MI could be yielded with a simplified setup, e.g., through at-home self-practice. However, state-of-the-art research suggests that the EEG-based control of the SMR-activation strength and consistency during an initial imagery habituation stage might still be a good idea. One alternative and potentially effective method of enhancing the learning and practice of motor imagery could be the addition of supplementary congruent sensory stimulation, exemplified in the present study by the “video-guided” imagery mode. Further research is required to identify the potential benefits of congruent stimulation for learning imagery in fully naïve subjects.

## Figures and Tables

**Table 1:** Estimates of fixed effects (*isFeedbacked:subj*) best linear unbiased estimators and related statistics for all well-known imagery conditions (FL, FR, SL and SR).

	Estimate (BLUE)	SEpred	CI 95%		tStat	DF	*p*-Value
			Lower	Upper			
*s01*	13.39	2.00	9.46	17.32	6.6879	631	5 × 10^−11^
*s02*	−3.61	2.06	−7.66	0.45	−1.7466	631	0.08119
*s03*	−1.53	2.09	−5.63	2.57	−0.7309	631	0.46510
*s04*	0.80	2.15	−3.41	5.02	0.3744	631	0.70825
*s05*	3.81	2.29	−0.69	8.30	1.6626	631	0.09689
*s06*	−0.59	2.06	−4.63	3.44	−0.2880	631	0.77342
*s07*	−2.61	2.17	−6.88	1.66	−1.2001	631	0.23055

## Data Availability

Raw EEG data and the derived metrics necessary for statistical inference available from the FigShare repository: https://doi.org/10.6084/m9.figshare.14602872.v2, accessed on 17 September 2021.
